# Superior Segmental Optic Nerve Hypoplasia: A Case Report and Literature Review

**DOI:** 10.7759/cureus.101687

**Published:** 2026-01-16

**Authors:** Minahil Mujahid, Muhammad Cheema, Husain Ahmed, Michael A Burdon

**Affiliations:** 1 Ophthalmology, Newcastle Upon Tyne Hospitals NHS Foundation Trust, Newcastle Upon Tyne, GBR; 2 Ophthalmology, South Tyneside and Sunderland NHS Foundation Trust, Sunderland, GBR; 3 Orthopaedics, Newcastle Upon Tyne Hospitals NHS Foundation Trust, Newcastle Upon Tyne, GBR; 4 Neuro-Ophthalmology, County Durham and Darlington NHS Foundation Trust, Durham, GBR

**Keywords:** ganglion cell layer, gestational diabetes, optical coherence tomography, retinal nerve fibre layer, ssonh, superior segmental optic nerve hypoplasia

## Abstract

Superior segmental optic nerve hypoplasia (SSONH) is a rare congenital anomaly in which there is underdevelopment of the superior part of the optic nerve. This leads to a reduction in the total nerve fibres constituting the optic nerve and can result in inferior visual field loss. SSONH is classically associated with maternal type 1 diabetes mellitus. We report an atypical case of SSONH associated with maternal gestational diabetes but with no prior history of maternal type 1 diabetes. We comment on the associated retinal nerve fibre layer (RNFL) changes, and also the corresponding ganglion cell layer (GCL) changes, which have not previously been explored using modern optical coherence tomography (OCT) macular segmentation. We discuss previous case reports on SSONH and outline the associated conditions. This review highlights the importance of correct and prompt diagnosis of this rare condition to avoid unnecessary investigation and undue long-term follow-up.

## Introduction

Superior segmental optic nerve hypoplasia (SSONH) is a rare congenital abnormality where there is underdevelopment of the superior part of the optic nerve, resulting in a reduction in total nerve fibres constituting the optic nerve [1]. This can lead to subsequent inferior visual field loss of a moderate to severe degree, meaning loss of vision affecting the lower half of the visual field [1]. It is important to note that SSONH is distinct from global optic nerve hypoplasia (GOH) [1-3]. Although the factors linked to the development of both conditions remain unclear, they are associated with different risk factors.

The classic risk factors associated with SSONH include maternal type 1 diabetes mellitus (T1DM) [4,5], paternal ischaemic heart disease (IHD) [4], female sex, and positive family history [1,2]. Conversely, GOH is associated with a multitude of conditions and is one of the principal features of septo-optic dysplasia and central nervous system malformations [1]. GOH is also associated with endocrine abnormalities, including growth hormone deficiency, hypothyroidism, hypocortisolism, panhypopituitarism, and hyperprolactinaemia. Literature has shown a strong association of SSONH with prior maternal T1DM. However, several case reports now describe SSONH occurring without this [6,7]. Our case report is unique and reports the presence of gestational diabetes only.

The prevalence of SSONH has been evaluated primarily in the Far East. A Korean study estimated its prevalence to be 0.24% in 5,612 subjects [4], while a Japanese study, evaluating prevalence by looking at fundus photographs, found it to be 0.3% among their subjects and 0.2% in the general population [8].

The exact aetiology of SSONH is unclear, although several associations have been reported. We summarise the published literature to outline the associations in previous case reports. We also report a unique case of SSONH with gestational maternal diabetes and preterm birth as a possible risk factor. Using modern optical coherence tomography (OCT), a non-invasive imaging technique that provides high-resolution cross-sectional views of retinal layers, we demonstrate superior thinning of the ganglion cell layer (GCL) and retinal nerve fibre layer (RNFL) associated with SSONH.

## Case presentation

A 25-year-old Caucasian male was referred by his optician after a routine eye test picked up an inferior altitudinal visual field defect in the left eye. The patient was initially referred to ophthalmology for glaucoma investigations. He was entirely asymptomatic with no visual symptoms, headaches, history of trauma, or past medical history. Family and birth history revealed that his mother had gestational diabetes, and he was born prematurely. There was no preceding history of maternal type 1 diabetes, as is commonly reported in literature [4,5].

**Figure 1:**
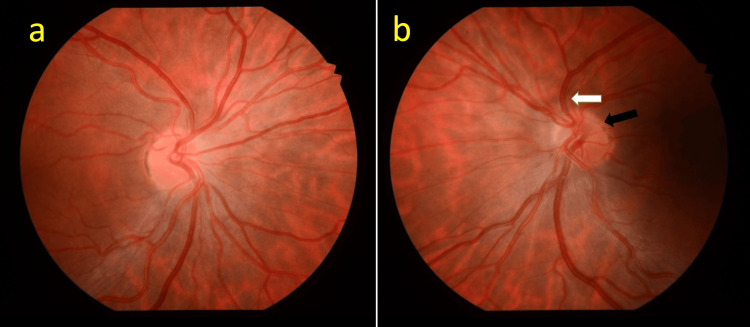
Fundus photographs of the right and left eyes at presentation. (a) Right optic disc with normal appearance of optic nerve and no evidence of hypoplasia for comparison. Cup: disc is 0.1, and there is a healthy 360° neuroretinal rim. (b) Left optic disc with superior segmental optic nerve hypoplasia (SSONH). There is the characteristic superior peripapillary “double-halo” sign (black arrow) and superior central retinal artery insertion (white arrow).

**Figure 2:**
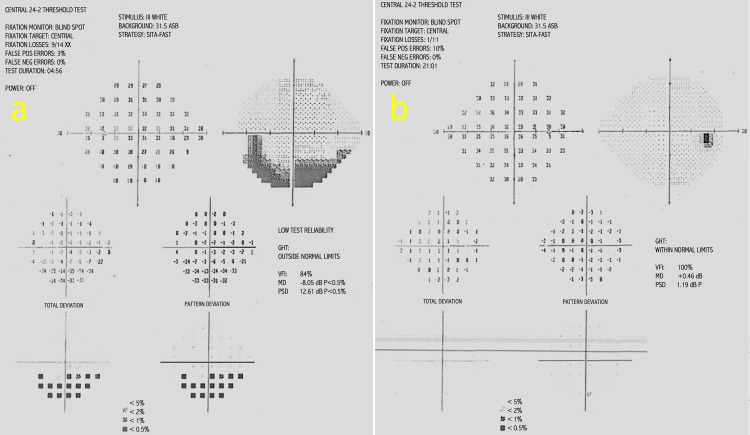
Humphrey’s 24-2 visual fields of left and right eye at presentation. (a) Left eye demonstrates an inferior altitudinal visual field defect. The greyscale plot shows dense inferior depression, with total and pattern deviation maps indicating defects in the same region. The glaucoma hemifield test (GHT) is outside normal limits, the visual field index (VFI) is reduced, the mean deviation (MD) is markedly negative, and the pattern standard deviation (PSD) is elevated, all of which correspond to the inferior field changes shown. (b) Right eye demonstrates a normal result with VFI, MD, and PSD within expected limits.

**Figure 3:**
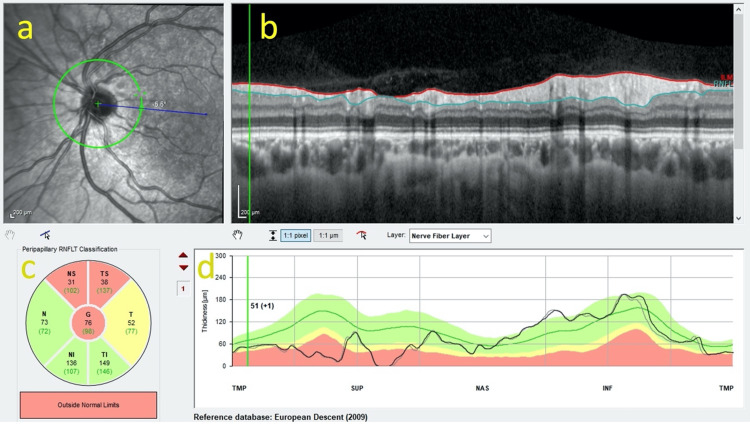
Optical coherence tomography (OCT) of the left optic nerve. Heidelberg OCT images of the left eye show marked thinning of the superotemporal and superonasal retinal nerve fibre layer (RNFL). (a) Peripapillary near-infrared fundus image showing the RNFL scan circle, centred on the optic nerve head. The crosshair marks the scan centre, and the reference line (blue) indicates scan angle. (b) Cross-sectional OCT B-scan through the peripapillary retina, with segmentation lines outlining the internal limiting membrane (ILM) and the outer border of the RNFL. (c) Sector-based RNFL thickness classification map (TSNIT sectors: temporal-superior, nasal-superior, nasal, nasal-inferior, temporal-inferior, temporal). Colours denote comparison with normative database: green = within normal limits; yellow = borderline; and red = outside normal limits. Each sector contains the measured RNFL thickness for the patient (black) and age-adjusted normative mean (green, brackets). The centre ‘G’ value represents the global mean RNFL thickness. (d) Thickness profile graph displaying the TSNIT RNFL curve. The patient’s RNFL profile (black line) is plotted against normative reference ranges (green/yellow/red bands).

**Figure 4:**
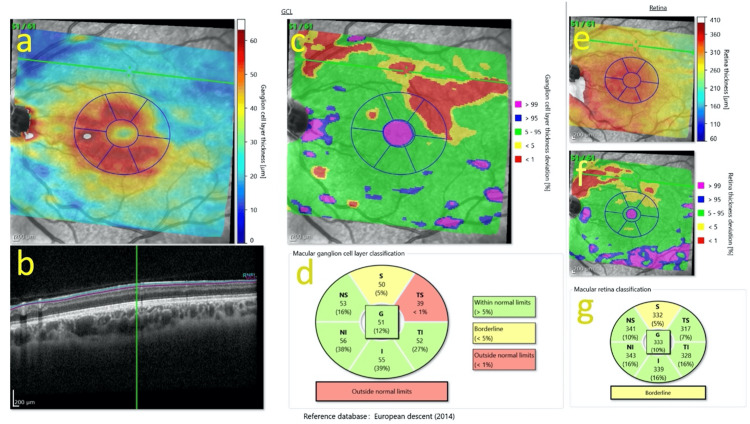
Multi-modal ocular coherence tomography (OCT)-based macular analysis of the left optic nerve. Heidelberg images of the ganglion cell layer (GCL) in the superior segmental optic nerve hypoplasia (SSONH) disc, with superotemporal thinning of the GCL. (a) Infrared fundus image with overlaid macular thickness topography showing absolute GCL thickness. There is an overlaid peripapillary scan ring. (b) Horizontal macular OCT B-scan through the fovea outlining retinal nerve fibre layer (RNFL) and GCL. The position of the B-scan on the en-face images is marked (green line). (c) GCL deviation map. The thickness of each point is compared to an age-matched normative database. (d) Macular GCL sector thickness classification plot. Sectors (TSNIT: temporal-superior, nasal-superior, nasal, nasal-inferior, temporal-inferior, temporal) display the mean ganglion cell layer thickness in micrometres and the corresponding percentile from the device’s age-matched normative database. The central ‘G’ value denotes the global mean GCL thickness. Colour coding reflects comparison with the normative database: green = within normal limits; yellow = borderline; and red = outside normal limits. (e) Macular RNFL thickness map showing absolute RNFL thickness in micrometres across macular subfields. The en-face image of the optic nerve head is overlaid with a colour-coded thickness map and a circular peripapillary scan ring. (f) Macular RNFL deviation map illustrating RNFL thickness deviation level relative to an age-matched normative database. (g) Macular retina classification plot. The macula is divided into the same central and peripheral sectors as in panel (d), with the same colour-coded comparison to a normative database. Within each sector box, the number indicates the mean retinal thickness in micrometres, and the percentage denotes the percentile for that sector relative to the reference distribution.

On examination, he had a left eye grade 1 relative afferent pupillary defect (RAPD) and normal Ishihara colour plates in both eyes. Best-corrected visual acuity was 6/6 in both eyes. Fundus examination of the left eye showed superior segmental hypoplasia of the optic nerve, with relatively superior entry of the central retinal artery (CRA) into the nerve and an almost negligible cup present (Figure 1B). The right eye examination was normal (Figure 1A). There was also superior segmental pallor, superior peripapillary halo and concurrent peripapillary nerve fibre layer thinning. Humphrey’s 24-2 visual field test showed an inferior altitudinal visual field defect in the left eye (Figure 2A). Optical coherence tomography (OCT) of the optic disc showed superotemporal and superonasal thinning of the RNFL (Figure 3), and macular segmentation studies showed GCL thinning in the superotemporal region (Figure 4).

**Figure 5:**
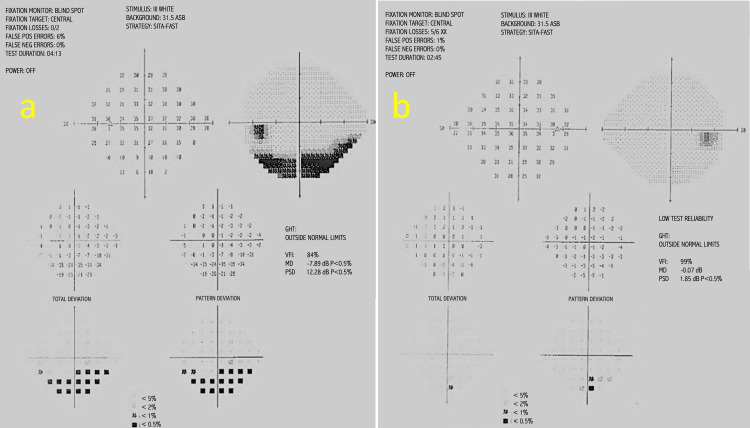
Humphrey’s 24-2 visual fields of left and right eye at follow-up. Repeat Humphrey’s 24-2 visual fields at nine-month follow-up show stable visual field results in the (a) left eye and (b) right eye, as compared to the initial examinations (Figure 2).

The patient’s visual fields were repeated nine months later and showed no evidence of progression and static features (Figure 5A). The right eye examination remained normal (Figures 2B, 5B). Repeat OCT RNFL scans also showed stable superotemporal and superonasal thinning in the left optic nerve.

In summary, the diagnosis of SSONH was supported by the presence of a characteristic optic disc appearance with superior peripapillary changes, a corresponding inferior altitudinal visual field defect, and concordant superotemporal and superonasal RNFL and GCL thinning on OCT, with stability on follow-up.

## Discussion

Subtle optic nerve head (ONH) findings play an important role in recognising SSONH, as the characteristic changes may be easily overlooked or misattributed to glaucomatous or anatomical disc variation. In our case, structural imaging showed a clear correspondence between the areas of RNFL thinning and GCL loss, with both changes localised to the superotemporal region. This anatomical concordance suggests underdevelopment of retinal ganglion cells (RGCs) and their axons in the same quadrant. It also supports the diagnosis of a congenital, rather than acquired, optic nerve anomaly. Notably, GCL involvement in SSONH has been only sparsely described in previous reports; our findings contribute additional structural detail characterised with modern macular segmentation OCT (Figure 4). The stability of the patient’s visual fields and OCT measurements over nine months further reinforces a non-progressive, developmental origin. Recognising these patterns, alongside relevant perinatal history such as maternal gestational diabetes, is essential in distinguishing SSONH from glaucomatous and other pathological causes of optic nerve asymmetry. This can prevent unnecessary investigations and prolonged follow-up.

These findings can be further contextualised by examining previously reported cases of SSONH, which demonstrate a range of perinatal and systemic associations (Table 1). SSONH was initially reported as a unique disc abnormality by Petersen et al. [2]. He described 17 cases of segmental optic nerve hypoplasia associated with maternal diabetes [2]. Subsequently, Nelson et al. established more defined characteristic features [5]. The condition was later coined superior segmental optic nerve hypoplasia by Kim et al. [9].

**Table 1:** Literature review of case reports and prevalence studies describing associations with superior segmental optic nerve hypoplasia (SSONH).

Report	Number of cases/total	Unilateral (U/L) or bilateral (B/L)	Maternal diabetes	Other associations
Petersen et al. [2] (1977)	17/93	14 – B/L, 3 – U/L	Yes	Preterm birth
Nelson et al. [5] (1986)	4	2 – B/L, 2 – U/L	Yes	Preterm birth, sensorineural hearing loss, low birth weight
Kim et al. [9] (1989)	10	7 – B/L, 3 – U/L	Yes	Unknown
Brodsky et al. [1] (1993)	2	B/L	Yes	Normal birth weight
Landau et al. [10] (1998)	3/34	1 – U/L, 2 – B/L	Yes	Preterm birth
Hashimoto et al. [6] (1999)	4	1 – B/L, 3 – U/L	No	Japanese cohort, normal term babies
Purvin [11] (2002)	1	B/L	Yes	No associations
Unoki et al. [12] (2002)	7/10	2 – B/L, 3 – U/L	3 – Yes, 2 – No	No associations
Yamamoto et al. [8] (2004)	54/28,396	17 – B/L, 20 – U/L	Unknown	Unknown
Foroozan et al. [13] (2005)	1	B/L	Yes	Normal birth weight
Ohguro et al. [14] (2008)	1	B/L	No	No associations
Sowka et al. [15] (2008)	1	U/L	No	Unknown
Han et al. [7] (2009)	3/3905	U/L	No	No associations
Kim et al. [16] (2012)	1	U/L	No	Unknown
Seo et al. [4] (2014)	14/5,612	2 – B/L, 12 – U/L	Yes	Paternal ischaemic heart disease
Shew et al. [17] (2018)	1	B/L	Yes	History of head trauma
Matos et al. [18] (2020)	1	U/L	Unknown	Preterm birth

Pathophysiology

Factors behind the development of SSONH still remain unclear. It has been postulated that optic nerve hypoplasia occurs due to either primary failure of RGCs or excessive apoptosis [10]. However, RGCs share the same progenitors as other retinal tissue cells, such as amacrine cells, which are formed at the same time as RGCs [19]. Therefore, it is unclear why other retinal tissue may develop normally despite RGC loss. Maternal diabetes has also been linked to the development of SSONH [2]. It has been shown that excitatory neurons in the RGCs are more susceptible to cell death when exposed to high glucose mediums [19]. This may be one plausible explanation as to why the RGCs may be more affected in maternal diabetes.

Clinical features and OCT findings

The four characteristic SSONH features are (1) superior entrance of the CRA, (2) superior peripapillary halo, (3) thinning of the superior peripapillary nerve fibre layer, and (4) superior optic disc pallor [9]. It is worth noting that in Asian populations, the superior optic disc pallor can be absent, and superior peripapillary thinning is primarily localised to the superonasal rim, thereby resembling a glaucomatous optic neuropathy [3]. Our patient demonstrated characteristic features of superior entrance of CRA, superior peripapillary halo, and thinning of the superior peripapillary nerve fibre layer. There was no superior optic disc pallor noted. There is also evidence showing greater extension of the retinal pigment epithelium over the optic disc with SSONH [20]. However, this tool to differentiate SSONH from normal optic nerves is still in developmental stages.

OCT findings in SSONH typically demonstrate RNFL thinning in the superior and superonasal quadrants [12,20]. In our case, RNFL reduction was accompanied by relative thinning of the superotemporal GCL sector (Figure 4D). However, this region did not fall outside normal limits on the GCL deviation map (Figure 4C). This difference reflects the analytical approaches used: deviation maps assess point-by-point focal abnormality against a normative database, whereas sectoral maps present the mean values across a broader, predetermined anatomical region. Consequently, the superotemporal sector is relatively thin within this eye, but not focally abnormal when compared to population-based norms.

Differential diagnosis of SSONH

SSONH is commonly misdiagnosed as normal tension glaucoma (NTG) due to abnormal, suspicious disc appearance and altitudinal visual field defects in the presence of normal intraocular pressure (IOP) [3,20]. This is even more significant in Far-East Asian populations, where the prevalence of NTG increases to 3.6% in those over 40 years old [3]. Especially considering the common absence of characteristic superior optic disc pallor in Asian populations, the chances of misdiagnosis as NTG are even more likely [3]. Therefore, it is crucial to be aware of the differences in presentation and background to differentiate SSONH from NTG, as both may have normal IOPs.

Features indicating more towards SSONH will be superonasal RNFL loss (particularly in Asian populations) and a corresponding visual field defect connected to the blind spot [14,18,20]. In NTG, primary rim thinning occurs in the inferotemporal and superotemporal quadrants in early stages [3]. This helps differentiate SSONH from NTG based on clinical features and visual field defects, especially in the early stages of NTG. However, clinical history and follow-up would support any conclusions made.

Other diagnoses to consider as differentials for bilateral or unilateral SSONH include trauma, anterior ischaemic optic neuropathy, coloboma, choroiditis, retinal detachment, bilateral occipital infarcts, and branch retinal artery occlusions [2,10].

## Conclusions

SSONH is a congenital optic nerve anomaly with a wider range of perinatal associations than traditionally recognised. Although maternal T1DM is the most frequently reported factor, this case demonstrates that SSONH may also occur in the setting of gestational diabetes and preterm birth. Recognition of its characteristic structural features, such as superior RNFL thinning, alongside less commonly described features, such as corresponding GCL loss, is essential to avoid misdiagnosis, particularly with normal-tension glaucoma. As multimodal imaging becomes increasingly available in both community and hospital eye care, improved awareness of SSONH can help reduce unnecessary referrals, investigations, and follow-up for patients with this non-progressive condition.
